# Micro-CT scan with virtual dissection of left ventricle is a non-destructive, reproducible alternative to dissection and weighing for left ventricular size

**DOI:** 10.1038/s41598-020-70734-3

**Published:** 2020-08-17

**Authors:** Ata Doost, Alejandra Rangel, Quang Nguyen, Grant Morahan, Leonard Arnolda

**Affiliations:** 1 Australian National University Medical School, Canberra, ACT Australia; 2 Illawarra Health and Medical Research Institute (IHMRI), University of Wollongong, Building 32, Wollongong, NSW 2522 Australia; 3 Centre for Diabetes Research, Harry Perkins Institute of Medical Research, University of Western Australia, Perth, Australia

**Keywords:** Cardiovascular diseases, Cardiovascular biology, Biological techniques, Anatomy, Cardiology

## Abstract

Micro-CT scan images enhanced by iodine staining provide high-resolution visualisation of soft tissues in laboratory mice. We have compared Micro-CT scan-derived left ventricular (LV) mass with dissection and weighing. Ex-vivo micro-CT scan images of the mouse hearts were obtained following staining by iodine. The LV was segmented and its volume was assessed using a semi-automated method by Drishti software. The left ventricle was then dissected in the laboratory and its actual weight was measured and compared against the estimated results. LV mass was calculated multiplying its estimated volume and myocardial specific gravity. Thirty-five iodine-stained post-natal mouse hearts were studied. Mice were of either sex and 68 to 352 days old (median age 202 days with interquartile range 103 to 245 days) at the time of sacrifice. Samples were from 20 genetically diverse strains. Median mouse body weight was 29 g with interquartile range 24 to 34 g. Left Ventricular weights ranged from 40.0 to 116.7 mg. The segmented LV mass estimated from micro-CT scan and directly measured dissected LV mass were strongly correlated (R^2^ = 0. 97). Segmented LV mass derived from Micro-CT images was very similar to the physically dissected LV mass (mean difference = 0.09 mg; 95% confidence interval − 3.29 mg to 3.1 mg). Micro-CT scanning provides a non-destructive, efficient and accurate visualisation tool for anatomical analysis of animal heart models of human cardiovascular conditions. Iodine-stained soft tissue imaging empowers researchers to perform qualitative and quantitative assessment of the cardiac structures with preservation of the samples for future histological analysis.

## Introduction

Left ventricular (LV) mass has been shown to be prognostic of cardiovascular morbidity and mortality irrespective of traditional risk factors^1–3^. Estimation of LV mass can be performed by several imaging modalities including echocardiography, cardiac computerised tomography scan (CT scan) and cardiac magnetic resonance (CMR)^4^. Echocardiography has been traditionally established as the most widely used diagnostic tool despite inherent limitations^4^. CMR is accurate and without risks of radiation but its availability, cost and patient tolerance make it an unattractive tool for clinical use^5^. Despite radiation and use of contrast agents, cardiac CT scan is another viable option for assessment of LV mass with high spatial resolution^6^.

Cardiac imaging in mice as opposed to humans poses problems that arise chiefly from their small size and fast heart rates^7^. Variable results have been reported with CMR in mice^8–10^ where fast heart rates pose a challenge and cost limits availability. Radiation exposure is not a significant impediment to studying mouse hearts but fast heart rates remain a challenge^11^. *Ex-vivo* measurements of mouse tissue are more easily implemented and have been evaluated here.

Our aim was to develop a non-destructive method of estimating *ex-vivo* left ventricular mass/tissue-volume in mice as part of a project aimed at identifying genes that contribute to left ventricular hypertrophy in the Collaborative Cross (CC)^12^. Various CC strains have been housed in different locations and have been moved through their history. The initial breeding sites^13^ were in Oak Ridge (USA), Perth (Australia) and Nairobi (Kenya) and these populations have moved to Chapel Hill^13^ (USA), Beijing (China) and Tel Aviv^13^ (Israel) respectively. This has created some difficulty with access to the strains for phenotyping. The ability to study the CC strains using fixed tissue allows access to more strains at a greatly reduced cost with fewer regulatory impediments compared to shipping live animals. Additionally, the use of a non-destructive method allows subsequent analysis by other methods including histological examination. Therefore, we evaluated the reliability of staining enhanced micro-CT scan for measuring the LV tissue–volume/mass in the mouse.

We applied staining enhanced micro-CT scan for high resolution imaging of cardiovascular structure in laboratory mice to reconstruct the LV and measure the LV tissue–volume/mass. Micro-CT is one of a number of new tomographic techniques that have revolutionised the study of embryogenesis by permitting the viewing and analysis of complex 3-dimensional structures had previously been studied by analysis of serial 2-dimensional histological sections^14–16^. The applications of micro-CT have included quantitative volumetric analysis at very small scale^16^ and the detailed geometric assessment of fine cardiac structures^14,15^. Virtual dissection of micro-CT 3-dimensional images provides non-destructive measurement of left-ventricular tissue volume allowing both secondary uses and repeated measurements neither of which are possible with physical dissection. Estimated LV mass was compared with the directly measured LV mass after dissection as this has been the common comparator in many imaging studies although, for small volumes, histomorphometry has occasionally been used^17^. Our results support the use of micro-CT to estimate LV tissue volume, particularly in circumstances where it is desirable to leave the preserved specimen intact for other subsequent use.

## Results

Thirty-five mouse hearts were included in this study. Mice were of either sex and 68 to 352 days old (median age 202 days with interquartile range 103 to 245 days) at the time of sacrifice. Samples were from 20 genetically diverse strains including 18 strains of The Collaborative Cross^18^. Median mouse body weight was 29 g with interquartile range 24 to 34 g.

### Differences between LV mass estimated from micro-CT (virtual dissection) and directly measured, dissected LV mass (physical dissection)

**Figure 1:**
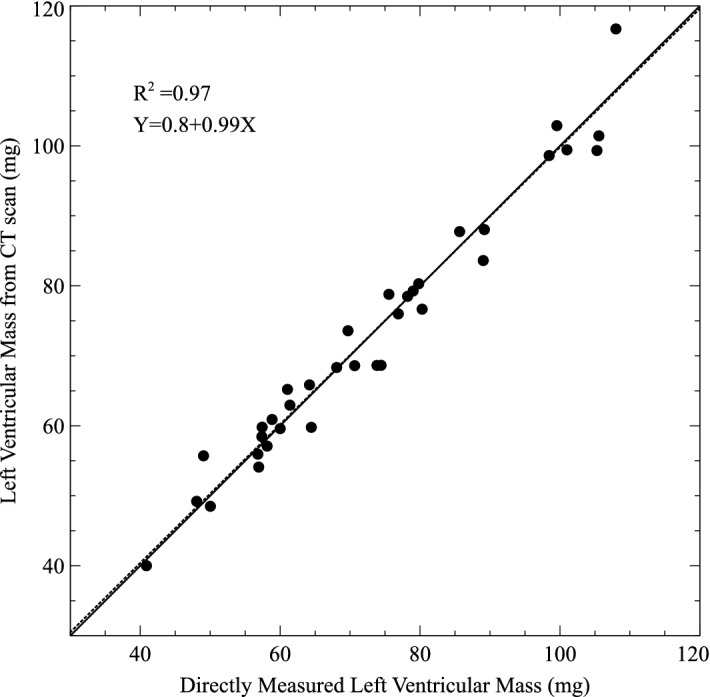
Scatter plot of left ventricular (LV) mass estimated from virtual dissection of micro CT versus directly-measured, dissected LV mass (median of 3 measurements). The solid line is the line of equality. The dashed line is a plot of the linear regression equation.

A scatter plot of estimated LV mass estimated from micro-CT and the measured LV mass is shown in Fig. 1. The two measures are closely correlated (R^2^ = 0.96). Individual paired measurements lie close to and on either side of the line of identity with a slope that is close to 1.

**Figure 2:**
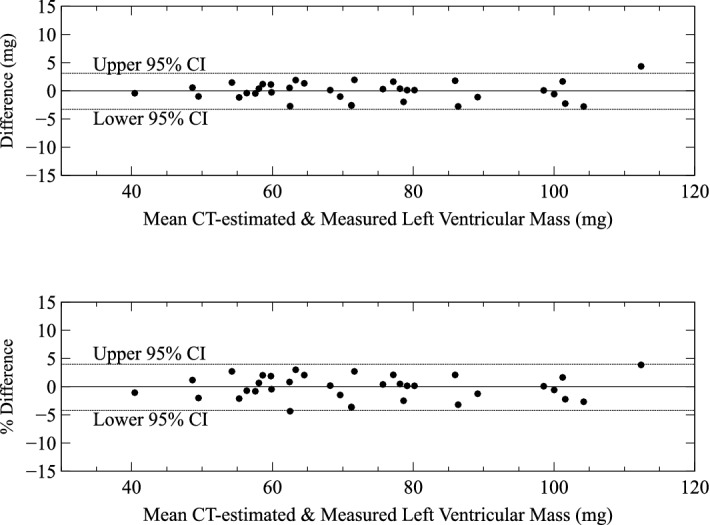
Difference between CT-estimated (virtual dissection) and directly-measured LV mass (physical dissection) versus mean left ventricular mass from the two methods. The upper panel shows the difference in grams and the lower panel the difference as a percentage of LV mass. *CI* confidence interval.

Figure 2 shows the absolute and percentage differences between LV mass estimated from virtual dissection of CT images of whole heart plotted against the mean of the two measurements (virtual and physical dissection of LV). There was no significant bias. The 95% confidence interval for difference between virtual and physical dissection were − 3.3 mg to 3.1 mg for absolute weight and from − 4.2 to plus 4% of LV mass.

### Repeatability of virtual dissection

#### Quantitative analysis

**Figure 3:**
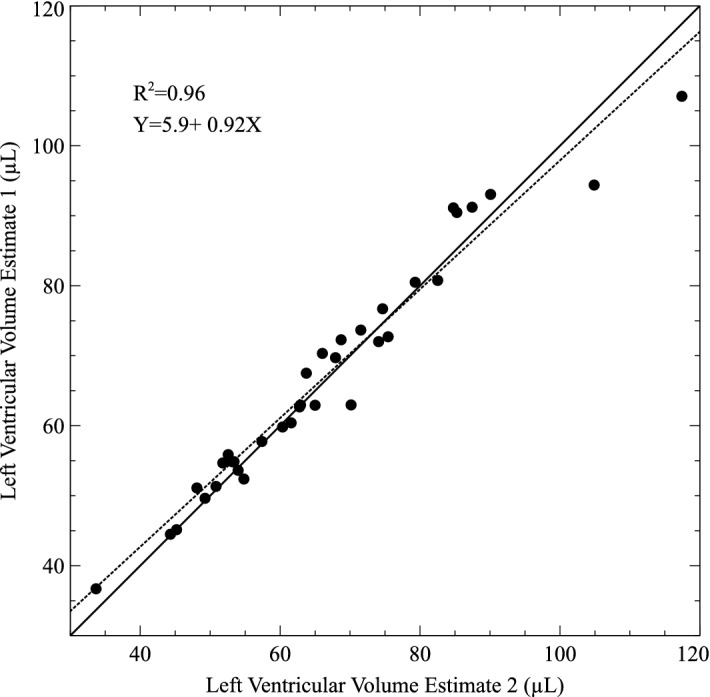
Scatter plot of independent virtual dissections of cardiac CT scans to yield paired repeated measurements of left ventricular tissue volume (μL). The solid line is the line of equality and the dashed line is the plotted linear regression equation.

A scatter plot of measurements of LV tissue volume by independent virtual dissections is shown in Fig. 3. Paired measurements are closely correlated. Individual measures lie close to and on either side of the line of identity with a slope that is close to 1.

**Figure 4:**
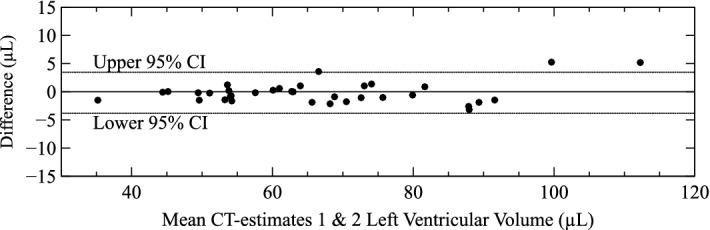
Difference between independent measurements of left ventricular tissue volume from virtual dissections of cardiac CT scans versus mean left ventricular tissue volume. *CI* confidence interval.

Figure 4 shows the difference in volume between independent measurements of LV tissue volume from virtual dissection of CT images of whole heart plotted against the mean of the two measurements. There was no significant bias. The 95% confidence interval for difference between repeated virtual dissection was − 3.8 µL to 3.5 µL.

#### Qualitative analysis

**Figure 5:**
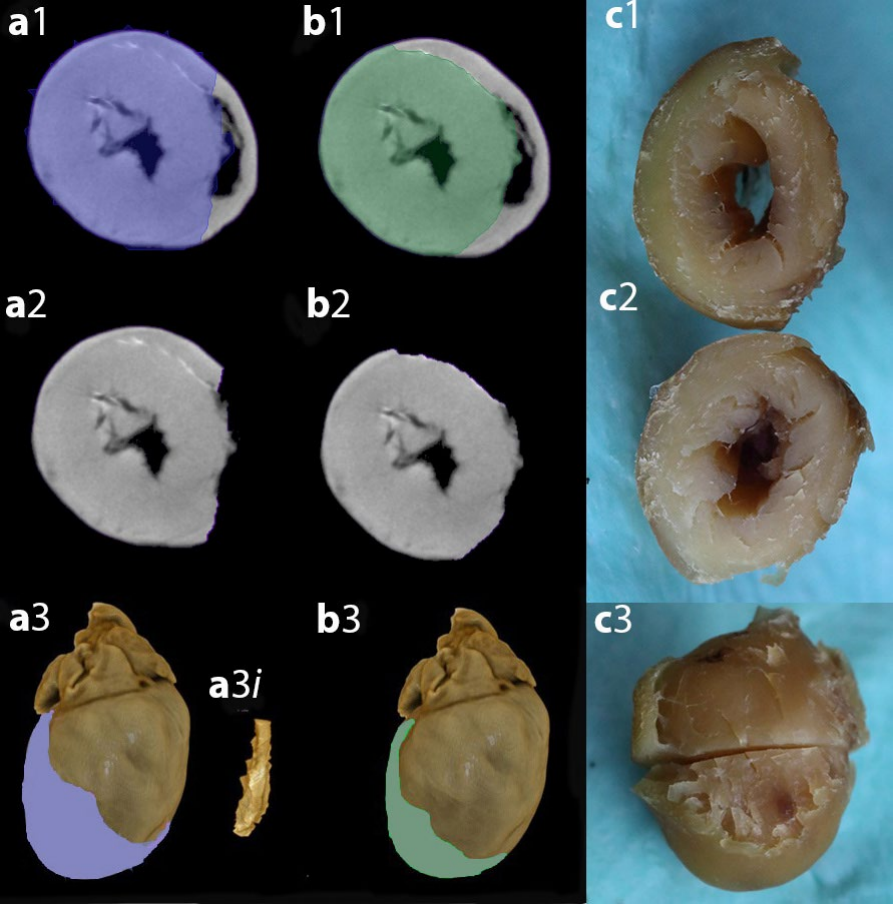
Images from one heart showing differences in Virtual dissection by observer 1 (**a**) and Observer 2 (**b)** and physical dissection (**c)**. Images were chosen to illustrate points made in the text rather than to represent average differences. Selections made by Observer 1 are labelled **a**1, **a**2 etc*.* Selections made by Observer 2 are labelled **b**1, **b**2 etc*.* Photographs of the physically dissected heart are shown in the right panel labelled **c**. Panel **a1** shows the region selected as being the left ventricle (LV) by Observer 1 (blue shaded area) and Panel **b1** shows the region selected as being the LV by Observer 2 (green shaded area). The area selected as being LV by Observer 1 (panel **a**2) was greater than the area selected as LV by Observer 2 (panel **b**2). Panels **a**3 and **b**3 show the three-dimensional structure of the heart with the unselected surfaces in brown and the selected areas in blue (Observer1, **a**3) or green (Observer 2, **b**3). Observer 1 and 2 show close agreement in removal of supra ventricular structures. The surface attributed to right ventricular free wall by Observer 1 is less than the surface attributed to right ventricular free wall by Observer 2. **a3i** shows LV clot segmented and removed by Observer 1. Photographs of cut surface of LV and base of heart **(c**1), cut surface of LV and apex of heart **(c**2) and the LV with cut surfaces apposed and the right ventricle removed exposing the septal surface of the RV (**c**3). Imprecision in physical dissection is apparent in the ragged edges where the RV free wall was removed.

There were some differences in the approaches taken by Observers 1 & 2. An obvious example demonstrating these differences is shown in Fig. 5. Observer 1 tended to take the shortest path from the margin of the RV septal surface to the epicardial surface whereas Observer 2 tended to follow the contour of the endocardial surface of the RV. In the example shown in Fig. 5 the area of LV selected by Observer 2 is about 89% of the area selected by Observer 1. Figure 5 shows differences between Virtual Dissection by Observer 1 (5a) & 2 (5b) over the RV surface. It is likely that a detailed protocol would attenuate these differences. Clot in the LV cavity (a3i) was excluded by Observer 1 but not by Observer 2.

### Repeatability of measurement of mass in physically dissected LV

**Figure 6:**
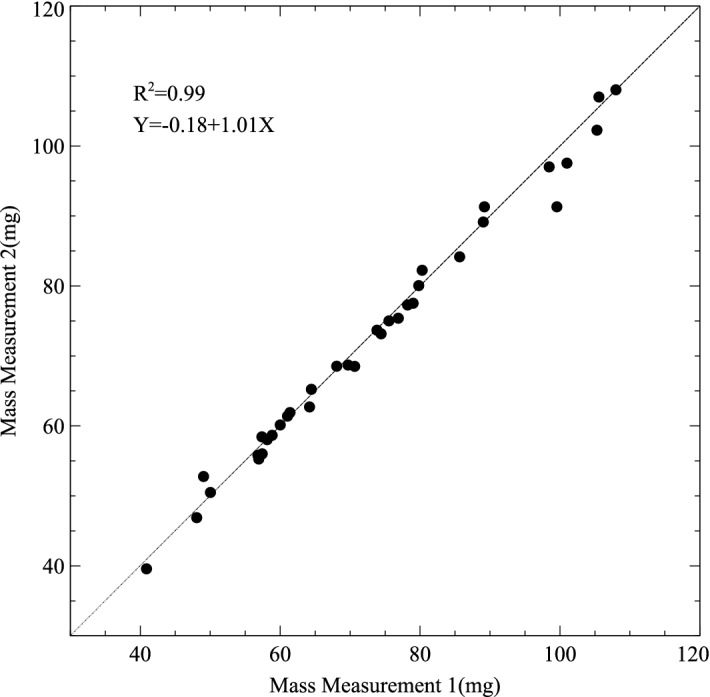
Scatter plot comparing repeated measurements of mass of a dissected left ventricle on an analytical balance. The line of equality (solid line) and the plotted regression equation (dashed line) are virtually superimposed.

**Figure 7:**
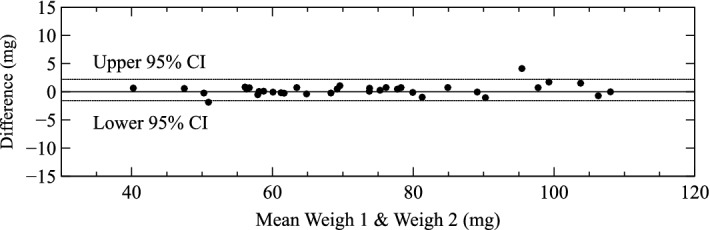
Difference between independent measurements on an analytical balance of LV tissue mass from a single physical dissection of the LV. Note this analytical error is likely a small part of the error associated with dissection of the heart for direct measurement of LV mass. *CI* confidence interval.

Repeated measurements were closely aligned to the line of identity with a narrow 95% confidence interval from − 1.6 to 2.2 mg (Figs. 6, 7). This measurement error was approximately 60% of the difference between Virtual Dissection (mass estimated from micro-CT) and Physical Dissection with weighing. The errors that arise from inaccuracy in Physical Dissection per se (Fig. 5c) cannot be easily measured since physical dissection is a destructive technique that cannot be repeated on the same sample.

## Discussion

We applied staining enhanced micro-CT scan for high resolution imaging of cardiovascular structure in laboratory mice to reconstruct the LV and measure the LV tissue–volume/mass. We have demonstrated that Virtual Dissection of X-ray micro-CT scans of iodine stained mouse hearts to determine LV size can replace physical dissection and weighing.

Micro-CT is one of a number of new tomographic techniques that have revolutionised the study of embryogenesis by permitting the viewing and analysis of complex 3-dimensional structures that previously been studied by analysis of serial 2-dimensional histological sections^14–16^. The applications of micro-CT have included quantitative volumetric analysis at very small scale^16^ and the detailed geometric assessment of fine cardiac structures^14,15^. Virtual dissection of micro-CT, 3-dimensional images provides non-destructive measurement of left-ventricular tissue volume allowing both secondary uses and repeated measurements neither of which are possible with physical dissection. Estimated LV mass was compared with the directly measured LV mass after dissection as this has been the common comparator in many imaging studies although, for small volumes, histomorphometry has occasionally been used^17^. Our results support the use of micro-CT to estimate LV tissue volume, particularly in circumstances where it is desirable to leave the preserved specimen intact for other subsequent use.

This non-destructive method lends itself to the assessment of repeatability that is not possible with physical dissection, provides a permanent record of the three-dimensional relationship of cardiac structures that others have used to identify cardiac defects^14,15^ and permits the use of cardiac tissue for other purposes. The micro-CT scanning protocol with iodine staining used here offers a simple, quick and inexpensive method to phenotype cardiovascular and other soft tissue structures in laboratory mice. Micro-CT has previously been widely used to study birth defects^2,16,19^ where its ability to provide a three-dimensional representation at very high resolution is unrivalled. Micro-CT can resolve structures that are much too small to be checked by physical dissection and weighing^20^. Thus, it would be inappropriate to regard dissection and weighing as providing a “gold standard” for assessing the accuracy of micro-CT determination of LV size.

Measuring LV mass is the traditional method of assessing left ventricular hypertrophy in large animals but it poses some problems when applied to very small mouse hearts. Removal of great vessels, atria and free wall of the right ventricle is time-consuming and difficult to complete accurately because of the small size of the organs. Thus, Ghanem et al.^17^ note, ‘For the assessment of left-ventricular mass (LVM) in mice histomorphometry used to be the “golden standard,” (sic) as necropsy and assessment of wet heart weight has been found to be inaccurate particularly in smaller mice’ and Franco et al.^7^ did not attempt to remove the free wall of the RV because “dissection of the free wall was prone to error”. Quantification of these errors is difficult as the method is destructive and cannot be repeated on the same heart. The limits of determination of volume by micro-CT is readily quantified^20^ and the repeatability in practical applications can be measured.

Micro-CT scanning compares very well with existing methods for determining LV mass. The level of agreement we noted between the micro-CT and measuring mass after dissection (95% CI − 3.3 mg to 3.1 mg or − 4.2% to plus 4%) is closer than most previously reported echocardiographic or MRI measurement of LV mass in live animals, shown in Table 1. Only a study by Dawson et al.^8^, using “*in house*” equipment and software, reported results that were close to ours in the level of accuracy for 3D echo (− 5.3 mg to 5.5 mg or − 8% to 8.3%) and MRI (− 5.6 mg to 4 mg or − 8.5 to 6%). The span of the 95% CI in other studies in Table 1 ranged from 30 to 106 mg.

**Table 1:** Mean with upper and lower 95% confidence limits for agreement between measured LV mass and mass in mg estimated by various imaging modalities.

Method	Author	Upper 95% CI	Mean	Lower 95% CI
M-mode echocardiography	Gardin^33^	− 11.0	− 47.7	− 84.5
	Collins^34^	91.6	38.4	− 14.8
	Kiatchoosakun^35^	38.9	13.1	− 12.7
	Donner^36^	18.1	4.5	− 9.1
2D echocardiography	Collins^34^	29	1	− 27
	Kiatchoosakun^35^	35.3	10.2	− 14.9
	Dawson^8^	6	− 10	− 36
3D echocardiography	Dawson^8^	5.5	0.1	− 5.3
Magnetic resonance imaging	Franco^9^	2.41	− 15.4	32.3
	Ruff^10^	17.0	0.093	− 18.3
	Dawson^8^	4.00	− 0.80	− 5.6
Micro-CT scan	This study	3.10	− 0.09	− 3.29

Values for Gardin are calculated from tabulated data in their paper. Values for Franco and Ruff are measured from the figures provided in their papers.

The percentage error compared to weighing (− 4% to 4%) was also very good in comparison with studies in humans where MRI is considered to provide a “gold standard”. Farber et al.^21^ compared MRI to explanted hearts obtaining levels of agreement from − 26% to 15% which is a much broader range than obtained in our study.

It is likely that the level of agreement we observed between micro-CT and dissection is close to the notional “repeatability” of dissection and weighing. The “error” in physical dissection is likely to be substantially larger than the error in determining mass on an analytical balance. The first error could only be assessed by simulation on a phantom whereas the latter error estimated by 95% CI for repeated determination of mass on the same piece of tissue was − 1.6 mg to 2.3 mg. This amounts to approximately 60% of the CI for the difference between micro-CT and dissection.

In our study repeatability was − 5% to 4% of LV mass. Moreover, our qualitative analysis suggests reproducibility could be improved by developing a more detailed protocol. Better repeatability and accuracy might also be achieved by using fully automated methods, for example by the implementation of Deep Learning for LV segmentation^22^. In the best of the in vivo animal studies Dawson et al. reported 95% CI for intra-observer repeatability from − 4 to 12% of LV mass determined by 3-D echo. Another study of repeatability of MRI^23^ reported 95%CI from − 8 mg to 11 mg (− 10.5% to 14%). Interstudy repeatability with MRI in humans was − 8% to 7% in a study by Grothues et al.^24^.

As an alternative to weighing, Ghanem et al.^17^ compared echocardiographic measurements of LV mass with serial sectioning at 500 μM intervals and 3D reconstruction of the LV. Micro CT scanning is conceptually similar to this approach of serial sections with computerised 3D reconstruction.

Micro-CT scan has several advantages over histomorphometry, considered the reference standard for analysis of cardiac development^17^, including being non-destructive to tissue, having short image acquisition time and high resolution. However, micro-CT scan has limitations such as radiation and need for use of a contrast agent to visualise soft tissues. Iodine enhances contrast by diffusion through tissue layers and binding to glycogen within muscle cells^25,26^ and increasing X-ray absorption. High resolution CT can provide information content comparable to that of conventional histological sectioning and staining^19^ with the additional benefit of providing 3-dimensional information.

## Conclusion

In conclusion, results obtained through the micro-CT scan and iodine staining provided high-quality morphological information at the micrometre scale. The ability to study fixed tissue that is more easily transported than live animals allows collaboration more easily with colleagues at distant locations to study unique collections of mice. The clear advantages of micro-CT scan images over histology include a less tedious sample preparation protocol, and less time-consuming without the need for sectioning samples which could impose risks of tissue destruction and artefacts. Moreover, data obtained by the micro-CT scan enables 3D visualisation of slices in multiple imaging planes. We have confirmed that analysis of micro-CT scan data can provide accurate measurements of cardiac chamber mass. This enables investigators to use an efficient and reliable method to assess cardiac structures of larger quantity in a shorter time period without destruction of tissues.

## Methods

### Sample preparation

Mice were from the Geniad Collaborative Cross Colony^18^ or from colonies held at ANU bearing a spontaneous mutation in the ALMS1 gene^27^. Hearts were harvested from mice studied under ANU-AEEC approved protocols A2013/30 (28 mice) and A2013/47 (7 mice). Tissue collection was performed after euthanasia under isoflurane anaesthesia. Heart and great vessels were excised and then fixed in a 4% formalin solution until imaging preparation.

Formalin-fixed hearts were then immersed in ethanol in order to remove formalin from heart tissue. To prevent a further and abrupt shrinkage of the fixed sample, each heart was subjected to a graded series of ethanol solutions beginning with 20% ethanol for 24 h, then 50%, 70% and 90% ethanol for 1 day each. Following this stage, hearts were submerged in the 1.5% iodine potassium iodide (I_2_kI) in 90% ethanol solution. Stained hearts then were imaged at 72 h. Samples were washed in absolute ethanol and then placed on the CT scanner.

### Image acquisition

A commercial Caliper Quantum FX micro-CT scanner system (www.perkinelmer.com) was used to scan the mice hearts. The tissue specimen is placed on a stationary loading dock of the scanner, positioning it between the rotating system of X-ray source and detector. The scanning time was pre-set at 3 min with the field of view (FOV) 10 mm in diameter, and the chosen mode of fine quality. Other setup parameters of Caliper Quantum FX micro-CT scanner include Voltage 90 kV, Capture Small, CT 200µA, Live 80uA. The maximum resolution achievable by this setting is 10 µm/voxel. The resultant images were stored as DICOM series in coronal, axial and sagittal views. Post processing of the raw DICOM data, reconstruction and visualisation were performed using the Drishti software package^28^ (Australian National University).

### Left ventricular volume assessment (virtual dissection)

The semi-automated edge-based segmentation technique^29^ was used in Drishti Paint, a function of Drishti, in order to separate the LV from the rest of the heart. Using Drishti Paint, left ventricular boundaries were manually delineated in 10–15 CT scan slices. Afterwards, Drishti Paint would collate the tagged slices and in-between slices to create a segregated left ventricle from the whole heart. The result was manually adjusted to ensure accurate definition of the cardiac contours. Segmented LV myocardial volume is calculated by the Drishti software in µL.

To standardise LV volume measurement between samples, multiple parameters were followed to minimise inter-sample measurement errors. The left ventricle was defined from the aortic ring at the base to the left ventricular apex. Interventricular septum and mitral valve papillary muscles were also included as part of the LV volume. The right ventricular (RV) free wall was excluded. Virtual Dissection was performed independently by two observers. Differences were examined quantitatively (see “Analyses” below) and qualitatively.

### Left ventricular mass calculation

LV mass was calculated from the product of ventricular muscle volume calculated by Drishti and specific gravity of heart muscle, estimated from the average of the directly measured weights and the average of the estimated volumes of all left ventricles.

### Physical left ventricular dissection

The LV was dissected from the rest of the mouse cardiac structure under magnification and the mass of the dissected LV was measured using a high-performance analytical balance, (Shimadzu AUW220D) paying careful attention to the operating environment as described in the manual. Three sets of measurements were made on different days and the median value was used for comparison. Conceptually, there are two sources of error in Physical Left Ventricular Dissection. The larger error is likely to be the mechanical dissection of a small organ manually under a dissecting microscope. Unfortunately, the destructive nature of this approach renders impossible the assessment of repeatability. The smaller error is likely to be the measurement error which was assessed by comparing the first two of three determinations of mass.

### Analyses

The principal methods employed were those described by Bland and Altman to quantify agreement between two methods of measurement^30,31^. Prior exploratory analysis included graphing the data in scatter plots and regression analysis^32^.

#### Left ventricular mass estimated by segmentation of whole heart CT images and weight of dissected left ventricle

Differences between paired values were plotted against their mean. The mean difference indicates the presence (or absence) of bias between the 2 measurements. We also plotted the 95% confidence interval (mean ± 1.96 standard deviations of the difference) to indicate the span within which 95% of differences between paired measurements would be expected to lie. When it appeared possible that either the mean difference or variability of the differences increased as the magnitude of the measurement increased we elected to plot the percentage difference^30^ against the mean of the 2 measurements rather than log transforming the values, as initially recommended^31^, because we believed that percentages were more likely to be intuitively comprehended by the reader than logarithms and their antilogarithms. Moreover, expressing the level of agreement as a percentage permits comparison with assessments made in larger hearts, for example, human hearts.

### Compliance with ethical practice

All tissues and animals used in this study were handled in compliance with The Australian Code for the Responsible Conduct of Research, 2007 and Australian National University Animal Experimentation Ethics Committee (ANU-AEEC).

## Data Availability

Data and material used for this manuscript are stored in our research office and are available on request.
